# Proceedings of the Suffolk District Medical Society

**Published:** 1880-12

**Authors:** 


### PROCEEDINGS OF THE SUFFOLK DISTRICT MEDICAL SOCIETY.

October 30, 1880. The meeting was called to order by Dr. Hodges, at 7:45. Sixty members present.

Dr. T. B. Curtis read a paper on the significance of frequent micturition.

Dr. White inquired whether Dr. Curtis could suggest any reason for the more frequent micturition during the night.

Dr. Curtis said that he could not; but it was a recognized fact. Contrary to what would be expected, there was an increased secretion during the night.

Dr. Cornell related a case of frequent micturition in a man seventy years old. He was obliged to rise as many as twenty times during the night. He used the catheter to empty the bladder and obtained great relief.

Dr. Ayer thought the increased frequency at night was due to nervous irritation, and found that in many cases he could relieve it by an anodyne remedy. The best that he had used was a mixture of the bromide and iodide of potassium in camphor water. It was not always successful, but gave relief in many cases.

Dr. Cornell inquired if these were cases with enlarged prostate.

Dr. Ayer said they were, and thought that the good was accomplished by allaying the general nervous irritation.

Dr. Harlow had found benefit follow the use of an opiate in some of his cases; the frequency being diminished. He inquired what was Dr. Curtis' treatment.

Dr. Curtis replied that the treatment depended on the cause. In many of the cases there was inability to empty the bladder without artificial means. The catheter must be used in these cases, and the patient taught to use it. In instituting the treatment, great care should be manifested, as at first cystitis is apt to be excited. He would advise the use of a soft rubber or a Mercier's catheter.

Dr. Lyman thought that the important practical point was to empty the bladder. He did not think it best to quiet it by an opiate. Physicians must be on their guard so as not to allow the bladder to fill, and to do this the catheter must be employed. Must not be deceived by the patients passing water, for they could do this, but could not get the bladder empty. He asked if there was any way of making a differential diagnosis when a fluctuating swelling was found above the pubes, in these cases, except by using the catheter.

Dr. Curtis thought there was not. The patient could pass urine easily, though the force of the stream may be diminished, and there may be frequent stops; but these were merely presumptive signs, the use of the catheter being the only reliable one.

Dr. Hodges said that in obstructive disease of the prostate one often failed to get relief by not using the right kind of a catheter. We often see patients passing a catheter for years, but still suffering from retention. The trouble was that they were using a silver or stiff catheter. A soft catheter should be used. They may have more trouble in passing it, but Mercier's was generally easily introduced.

Dr. Curtis said that in some old chronic cases the bladder became so changed in shape that it could not be emptied. The walls are thickened, lose their suppleness, and the floor becomes irregular.

*Aortic Aneurism.*—Dr. F. W. Draper presented a specimen of aneurismal dilatation of the arch of the aorta, with indirect hæmorrhage into the pericardium. The patient was a woman, sixty-five years old, spare and ill nourished. She was found dead in bed. So far as could be learned, she had not presented any symptoms referable to the heart or great vessels the day before her death; she had followed her usual pursuits, and had retired in customary health.

At the autopsy, the pericardium was found fully distended with mingled fluid and clotted blood to the amount of ten fluid ounces. The ascending portion of the arch of the aorta was symmetrically dilated along its convex border, the dilatation being inconsiderable, not above the size of a small lemon. At the upper portion of the arch, immediately to the right of the opening of the innominate artery, there was a transverse fissure in the intima of the aorta; the edges of the rent were quite regular, and their length was about one inch. The blood escaping through this rent, instead of passing directly upward and outward into the anterior mediastinum, took an indirect course downward and backward, dissecting the sheath of the artery away from the middle coat and, backing nearly an inch beyond the line of union of the pericardium with the aorta, reptured the pericardium and filled its sac. The rupture was one inch and a half long, with irregular edges, but in general direction parallel with the axis of the artery. The sheath of the artery adjacent to the rupture was extensively dissected from the subjacent coats by the effused blood.

The heart was hypertrophied to a moderate degree, although the aortic valves showed no change beyond very slight thickening of their free margins. The lining of the aorta, not only near the rupture, but above and below, showed distinct atheromatous degeneration, some of the plates being calcareous.

Both lungs were engorged. The other organs of the body presented nothing noteworthy.

Dr. Draper remarked that the rupture of an aneurism of such small dimensions must be infrequent, while hemorrhage into the pericardium from such a source was confessedly very rare.

Dr. E. M. Buckingham reported a case of poisoning by nutmegs. The patient had taken two nutmegs powdered and steeped in water, one of them at 9 A.M. and one at 1 P.M. No symptoms were observed until 6 P.M., when they appeared suddenly; the head seeming to swell and the extremities to become partially numb. Drowsiness soon followed, but on lying down there was an uncomfortable sensation at the heart, to avoid which she forced herself to walk about. Two grains of tartar emetic, prescribed by the next apothecary, were followed by vomiting at intervals all night. When seen in the morning there was no symptom that could be fairly attributed to nutmeg, except a little numbness, and that wore off in the course of the day. The literature of the subject is limited, but one or two nutmegs have produced drowsiness and delirium, and larger quantities are said to have been fatal.

Dr. White asked the result, saying that the oil of nutmeg was an irritant, and was used as a rubefacient.

Dr. Buckingham said the patient recovered. There was vomiting, but whether from the nutmeg or tartar emetic he could not say.

November 13, 1880. The meeting was called to order by Dr. Hodges at 7.50. Twenty members present.

Dr. H. D. Hicks reported a case of mitral disease with compensatory hypertrophy. After viewing the anatomy and physiology of the heart, he gave the history of the case as follows: The patient was a merchant fifty years old. His father died of old age; his mother is still living.

With the exception of the last few years his health has always been good. He has been an active business man, exposing himself for years to great fatigue and nervous excitement, doing everything under great nervous pressure. During the last few years he has been exposed to debilitating sickness, having suffered with typhoid fever, congestion of the lungs, and intermittent fever. He had palpitation of the heart for fifteen years. In the progress of his business he has been accustomed to put great strains upon his heart in running to catch trains, in ascending long flights of stairs, and, above all, in depressing the action of heart by the habit of escessive smoking. In November, 1878, just after his recovery from intermittent fever and congestion of the lungs, he ran one-third of a mile over a rough road, encumbered with a bag and his overcoat. When he reached his destination he was so exhausted, his heart beat so forcibly, and his respiration was so difficult that he was obliged to lie down on the platform of the car, where he remained an hour before he recovered. About a year afterwards, while at lunch, he was seized with a most agonizing pain in the centre of the sternum. This did not last a great while, but upon going back to his business he had another attack of pain over the region of the heart, so violent as to make him stagger, He went home immediately, perspiring profusely, and in the utmost agony. One week later he began to be short-breathed.

On January 1, 1880, after visiting the theatre, he had another attack, which began with a chill. His breathing was difficult, panting in character, and any attempt at lying down was accompanied by symptoms of suffocation. This continued for six days, when his feet and legs began to swell. He was all this time under the care of a homœopathic physician, who at the end of this time was discharged.

Under his new treatment by digitalis he improved so much as to be able to lie down in two days. About January 14th thoracentesis of the left chest was performed, and a large amount of fluid withdrawn. During these two weeks the digitalis had been given with good effect, but after the tapping he complained of greater dyspnœa than before, and also of violent palpitation of the heart. On suspicion that it might be caused by the digitalis, that agent was withdrawn, with relief both to the dyspnœa and the palpitation. On renewing the medicine the troubles immediately returned, and were relieved by withdrawing it again.

The physical examination showed mitral disease with compensatory hypertrophy, and the chief peculiarity of the case was the apparently contradictory action of the digitalis. This was explained by showing that when the drug was first given the hypertrophy was not sufficient to regulate the pulmonary circulation. The withdrawal of the fluid from the chest relieved the lungs, and then the digitalis, by increasing the force of the heart, caused the same condition of engorgement in the lungs which before it had relieved.

Dr. F. I. Knight said that the physical signs, a præsystolic followed by a diastolic murmur, mentioned by the reader, were very unusual. In the first place, the mitral præsystolic murmur was almost always of a rough, vibratory character, and not a blowing sound, as described by Dr. Hicks; in the second place, there was not sufficient force in the current of blood, when it began to flow from the auricle to the ventricle, to cause a murmur, and hence a murumr distinctly diastolic, that is, beginning with diastole, could usually be set down as due to regurgitation, pulmonic regurgitation being very rare.

In this case the possibility of the præsystolic being due indirectly to aortic regurgitation would naturally suggest itself. As pointed out by Flint, free aortic regurgitation may so float out the mitral vavle at the beginning of diastole that when auricular contraction takes place the blood coming from the auricle impinges on the mitral valve, and sets it into vibration, causing a præsystolic murmur.

This was hardly likely in Dr. Hick's case as the symptoms (dropsy etc.) were those of mitral rather than aoric disease.

The reader had done good service in calling attention to the indiscriminate use of digitalis in all cases of heart disease. It was indicated only when the heart was weak, and was decidedly prejudicial in predominant hypertrophy.

Dr. A. N. Blodgett asked if Dr. Hicks knew of actual disease of the heart produced by tobacco.

Dr. Hicks did not, but thought that it might act as a predisposing cause by weakening the heart, and thus rendering it more liable to disease.

Dr. H. I. Bowditch said that he had seen a great many cases of what he called tobacco heart, but never saw one with a valvular murmur. He had seen cases of valvular disease in persons who used tobacco. Considered that the action of tobacco was the same as tea and coffee; all caused functional trouble, but he had never seen a case of valvular disease which he could attribute to either. There is sometimes a slight blowing murmur heard that should not be regarded as due to mitral disease.

Dr. Knight thought that in case of disease with murmur the question of the effect of tobacco was not sufficiently considered. He thought that organic disease might result from prolonged functional disturbance.

Dr. Hicks did not regard the trouble in his patient as a direct result of his habits, but that the heart had been so weakened that when the strain came it could not stand it.

Dr. J. Homans presented an ovarian cyst which he had removed a week ago. The patient was a widow, thirty-two years old, and knew that she had had the tumor four years, though probably it was of much longer duration. It was of the multilocular variety, and weighed twenty-two pounds. The operation lasted twenty minutes, and there had been no outward symptoms since. She had had one hypodermic injection of one-sixth grain morphia the evening of the operation. Her temperature had reached 100° F. only once, and her pulse had been 100 once. In treating the pedicle, he both ties and burns with the cautery.

Dr. Barnes presented a number of round worms which he had found in the fæces of a hen.

Dr. Bowditch reported a case in order to derive some information in regard to the nomenclature of disease. The patient, a gentleman seventy-four years of age, generally enjoyed robust health. He has grown stout lately, which interferes somewhat with his walking. About three weeks ago he had not been well for a few days, but continued attending to his business. Being worse than usual one day, he was obliged to return home. He complained of feeling extremely debilitated, and was found to be suffering with great prostration, the pulse being very feeble. He also had slight pain in the right side. At the base of the right lung, behind, the respiration was found to be slightly roughened, and it was thought that he was going to have an attack of pleurisy or pneumonia. In a few days fine rales were heard at this place, which soon became coarser. There was no dullness except one day over a small spot in the middle of the back. Never any bronchial respiration nor bronchophony. No cough nor sputa. Pulse rarely over 80 or 90; temperature, taken regularly, varied from 99° F. in the morning to 100° F. at night. Never had any head symptoms.

Bowels were regular, having a healthy, well-formed discharge each day except once, when for twenty-four hours he had nausa accompanied by diarrhœa. As at this time the abdomen was a little flatulent and distended, it was thought that he had typhoid fever, but there were never any rose spots or enlargement of the spleen. Although there was some trouble in the lung, it was not pneumonia. From the first there were no symptoms which are common in typhoid fever, but his colleague called the disease typhoid pneumonia. He wanted to know why it should be called this, when neither disease was present. He thought that it was a misnomer, and rather should be called simple continued fever.

Dr. Hodges asked if anything had been noticed about the perspiration.

Dr. Bowditch replied that lately he had perspired profusely, so much so as to annoy him. It had been controlled by atropine and rubbing with a salted flannel.

Dr. Hodges asked if continued fever did not terminate in this way, and if typhoid is not rare at this age.

Dr. Homans thought that typhoid and other diseases are not seen in elderly people, because they have had them when they were younger.

Dr. Blodgett spoke of two cases of cancer in which China turpentine had been used. In the first, five hundred pills had been given without any appreciable effect on the disease. The second case had been treated in the same way. After taking between seventy-five and one hundred pills the patient vomited a mass resembling turpentine, which, on examination, it proved to be.

Dr. Bowditch said that he had had no experience with the drug, but a friend had told him that he thought he had seen benefit follow its use, although the disease was now returning.

Dr. Cornell asked if any physician who was an authority had ever seen any benefit following its use.

Dr. Blodgett said that Mr. John Clay, of London, introduced the drug, and claimed that he had cured cancer by its use.

Dr. Foster spoke of the differet temperatures in a heated room on a cold day. He had made some observations on the subject, and found that the temperature was different on the floor, half-way up, and at the ceiling. In some of the rooms he had found a difference of forty degrees between the top and bottom.

